# Unraveling neural pathways of political engagement: bridging neuromarketing and political science for understanding voter behavior and political leader perception

**DOI:** 10.3389/fnhum.2023.1293173

**Published:** 2023-12-21

**Authors:** Tuna Çakar, Gözde Filiz

**Affiliations:** ^1^Department of Computer Engineering, MEF University, Istanbul, Türkiye; ^2^Graduate School of Science and Engineering, Computer Science and Engineering PhD Program, MEF University, Istanbul, Türkiye

**Keywords:** political neuromarketing, political engagement, voter behavior, political leader perception, machine learning, model development

## Abstract

**Introduction:**

Political neuromarketing is an emerging interdisciplinary field integrating marketing, neuroscience, and psychology to decipher voter behavior and political leader perception. This interdisciplinary field offers novel techniques to understand complex phenomena such as voter engagement, political leadership, and party branding.

**Methods:**

This study aims to understand the neural activation patterns of voters when they are exposed to political leaders using functional near-infrared spectroscopy (fNIRS) and machine learning methods. We recruited participants and recorded their brain activity using fNIRS when they were exposed to images of different political leaders.

**Results:**

This neuroimaging method (fNIRS) reveals brain regions central to brand perception, including the dorsolateral prefrontal cortex (dlPFC), the dorsomedial prefrontal cortex (dmPFC), and the ventromedial prefrontal cortex (vmPFC). Machine learning methods were used to predict the participants’ perceptions of leaders based on their brain activity. The study has identified the brain regions that are involved in processing political stimuli and making judgments about political leaders. Within this study, the best-performing machine learning model, LightGBM, achieved a highest accuracy score of 0.78, underscoring its efficacy in predicting voters’ perceptions of political leaders based on the brain activity of the former.

**Discussion:**

The findings from this study provide new insights into the neural basis of political decision-making and the development of effective political marketing campaigns while bridging neuromarketing, political science, and machine learning, in turn enabling predictive insights into voter preferences and behavior.

## 1 Introduction

The landscape of political discourse in the 21st century is undergoing a profound transformation, characterized by a convergence of methodologies inspired by academic fields as diverse as marketing, neuroscience, or psychology. This represents a turning point in the field of political science, by allowing novel techniques to dissect and understand the intricate phenomenon of electoral behavior. Central to this evolution is the imperative to unravel the multifaceted nature of voter engagement, encompassing not only cognitive but also emotive dimensions with political leadership and party brands. As this interdisciplinary field, often termed “political neuromarketing” or “neuropolitics,” has attracted increasing attention over the last decade, it has also called for rigorous academic scrutiny. However, the exploration of this promising field is tempered by a series of ethical considerations, necessitating finding a delicate balance between potential benefits and potential risks.

As the evolutionary wheel of political marketing methodologies turns, it propels itself beyond the transient vortex of passing trends and embeds itself firmly within the complexities of contemporary political ecosystems. The convergence of neuromarketing and neurobranding confronts conventional research paradigms head-on, tendering insights into the neurobiological mechanics that orchestrate voter behavior. Yet, ethical considerations stand sentinel-like, a sentinel safeguarding the sanctity of the political realm as these methodologies traverse its hallowed precincts. Irrevocably entwined with this dynamic tapestry of political marketing stands the burgeoning role of branding within modern political campaigns. Political parties adroitly wield branding strategies reminiscent of their consumer-marketing counterparts, leveraging unique selling propositions, promises of brand fidelity, and meticulous image curation to sculpt voter perceptions and exert influence upon electoral choices. The fusion of political branding and the realm of neuromarketing extends an unprecedented vista upon the hitherto uncharted terrain of voter conduct at a neurophysiological level, a vista that beckons with transformative potential, irrevocably intertwined with ethical considerations that cannot be ignored (Farah and Gillihan, 2012).

### 1.1 Branding in marketing and in politics

The seminal contributions of Aaker (1997) laid the foundation for the concept of brand personality, originally within the context of marketing. The subsequent adaptation of this concept to the realm of political leadership, championed by scholars like Caprara et al. (2002), introduced the innovative notion that political leaders, as entities separate from their party affiliations, possess distinctive brand personalities capable of resonating with voters. Building on this, Needham (2005) extended the discourse by introducing the concept of “leader-centric” branding, a paradigm in which the leader’s brand supersedes that of the political party. The use of advanced neuroscientific methodologies, including functional Magnetic Resonance Imaging (fMRI) and Electroencephalography (EEG), in this domain has ushered in a new era of investigations. These neuroscientific tools provide a solid foundation for understanding the nuanced and often unconscious psychological mechanisms that underlie the intricate processes leading to voters’ decisions (Fisher et al., 2008; Vecchiato et al., 2011). Nonetheless, as underscored by Westen et al. (2006), the emotional dimensions intrinsic to political branding should not be underestimated. In an era where political leaders have morphed into “emotional brands,” eliciting potent affective responses that wield considerable sway over voting behavior, ethical complexities appear with increasing visibility (Murphy et al., 2008; Farah and Gillihan, 2012). This encroachment on the emotive spheres of political behavior necessitates a judicious navigation of ethical considerations to ensure that we, as voters, avoid succumbing to potentially manipulative tactics just similarly to the debated cases related to manipulations of the consumers or decision-makers (Ulman et al., 2015). Moreover, the interplay between political leadership and party branding defies simplistic cause-and-effect dynamics, instead manifesting itself within intricate neurocognitive frameworks that are influenced by cognitive biases, as postulated by Lau and Redlawsk (2001). The role of political leaders’ extends beyond that of mere figureheads; they can amplify or temper the party’s brand perception depending on the resonance of their individual traits with the electorate’s pre-existing perceptions of the party’s identity. Recent technological advancements, such as neuroimaging techniques, have made it possible to quantify these phenomena, offering the potential for more precisely targeted political marketing strategies that are both effective and ethically defensible.

Within liberal Western democracies, political marketing is undergoing a profound and transformative phase, driven by rapid advancements in technology and the burgeoning field of applied neuroscience. This transformative trajectory is intertwined with the reality of contemporary politics, where the ability to efficiently adapt to a rapidly evolving voting landscape has become a critical component of political survival and success. Conventional research methodologies, such as focus groups and surveys, are now complemented by a wide array of data analytics, neuroscientific investigations, machine learning algorithms, and predictive voter modeling. The intricate interplay between political leadership, party branding, and voter behavior is more pronounced than ever (Baines and Egan, 2001; Krieger and Zhang, 2014; Jungherr, 2016).

### 1.2 From traditional methods to neuroscientific methods

At the heart of the exploration of leadership perception stands to exert a discernible influence on voter conduct, the construction of party brand image, and the ultimate outcomes of electoral contests. Researchers have started exploring the roles played by media portrayals, campaign messaging, and the rapidly evolving digital and social media landscape, in shaping the public’s perception of political leadership (McCombs and Shaw, 1972; Parmelee and Bichard, 2011). The traditional research methodologies used by these scientists, however, have been criticized for their susceptibility to biases, inherent in self-reported data, which in turn has paved the way for the emergence of political neuromarketing, or neuropolitics—a subfield of the domain of applied neuroscience—that has the potential to deliver more objective and quantifiable measurements of voter response using tools such as functional Magnetic Resonance Imaging (fMRI) and Electroencephalography (EEG) (Fisher et al., 2010).

The combination of several neuroscientific tools, such as fMRI and EEG, has opened the door to a better understanding of the way political messages are processed in the human brain. They have afforded a deeper understanding of the emotional and cognitive mechanisms that guide voter decision-making (Falk et al., 2012). It is within this context that the concept of neurobranding has emerged, which considers the way branding elements such as campaign slogans and logos trigger neural responses and subsequently influence voter behavior (Vecchiato et al., 2011). The current evolution of political marketing, which is also related to the methodologies used, is more than a trend; it is a necessity in order to untangle the intricacies of modern political ecosystems. The combination of neuromarketing and neurobranding potentially presents a profound challenge to conventional research paradigms by offering new insights into the neuroscientific mechanisms that underlie voter behavior. However, the application of research tools to the domain of politics necessitates a robust and comprehensive ethical framework.

The understanding of the critical traits that shape voting behavior, such as trustworthiness, competence, and empathy, has been fundamentally transformed through the application of advanced neuroimaging techniques (Kato et al., 2009). These techniques not only serve to confirm the effectiveness of messaging strategies that accentuate such traits, but also enable the creation of meticulously tailored campaigns that target specific segments of the electorate. The extension of Jungian archetypes to the realm of political leadership branding, an endeavor informed by the principles of neuromarketing, also offers novel insights into how voters perceive the personas of political leaders (Zaltman, 2003). The relationship between political leadership and party branding is not superficial but profoundly embedded within the cognitive and emotional frameworks that guide voter behavior. It is within this landscape that the field of neuropolitics comes into play, shedding light on the neurocognitive mechanisms underlying cognitive biases, partisan loyalty, and phenomena such as the Halo and Horns Effects (Westen et al., 2006; Kanai et al., 2011). With the advent of neuroimaging methodologies, the exploration of the neuro-resonance between leaders and their party’s brand emerges, serving as a more objective metrics to gauge the alignment between leadership and party, and the concomitant impact on voter behavior (Harris et al., 2018).

### 1.3 Use of neuroscientific methods in political marketing

Understanding the attitudes of party members toward the leaders of their respective parties has taken a central place in contemporary academic literature. Using neuroimaging tools, this study aims at revealing the neural correlates that underlie responses to positive and negative trait adjectives. By discerning the variations in activation patterns in frontal brain regions among subjects affiliated with distinct political parties when exposed to images of political leaders alongside positive or negative adjectives, the study aims to improve our understanding of the intricate dynamics between leadership perception and party affiliations. While functional Magnetic Resonance Imaging (fMRI) has traditionally been the hallmark of such studies, here we chose to use the functional near-infrared spectroscopy (fNIRS) system due to its cost-effectiveness and its growing acceptance within scholarly circles (Botvinick et al., 2004; Kerns et al., 2004; Ridderinkhof et al., 2004; Van Veen and Carter, 2006).

Political decisions often exhibit a level of uncertainty regarding the eventual outcomes, a variable recognized to influence reward-related signals within the ventromedial prefrontal cortex (vmPFC), as evidenced in economic paradigms (Krastev et al., 2016). Moreover, a body of evidence stemming from human lesion studies underscores the role of the ventral frontal lobe, encompassing both the vmPFC and the orbitofrontal cortex (OFC), in shaping value-based choices across diverse contexts, including those within the realm of political decision-making (Krastev et al., 2016). This substantiates the assertion that the vmPFC is an indispensable neural substrate for value-driven decisions as illustrated with various empirical findings (Çakir et al., 2018). Political decision-making is often guided by the application of heuristics or cognitive shortcuts, as postulated by established scholarship (Popkin, 1991; Sniderman et al., 1993; Lupia, 1994). Citizens, in this regard, frequently draw cues from interveners and agenda-setters, evaluate the performance of the incumbent government based on economic indicators, consider a candidate’s party affiliation or ideological alignment, employ party affiliation as a proxy for issue positions, or even evaluate candidates based on superficial factors such as appearance or social background characteristics (Popkin, 1991; Rahn, 1993; Lau and Redlawsk, 2001). In contrast, political behavior delves into individuals’ preferences concerning aspects of societal organization and collective structure which may or may not directly impact them personally (Krastev et al., 2016). The ramifications of voting extend potentially to all members of a given society, and the outcome of the vote and its anticipated advantages hinge upon the choices made by fellow citizens.

Empirical findings from the literature reveal an increase in neural activation in response to statements that focus on individual interests, such as “Everybody should prioritize his or her own interests over society’s” (Zamboni et al., 2009). Conversely, statements emphasizing societal interests, like “Citizens should vote based on collective interest,” lead to heightened activation in the right dorsomedial prefrontal cortex (dmPFC) and left Temporoparietal Junction (TPJ). The degree of conservatism in individuals was linearly associated with the activation of the right dorsolateral prefrontal cortex (dlPFC), particularly in response to more conservative and stereotypical statements like “Everybody should oppose teaching evolutionary theory” (Zamboni et al., 2009). These findings suggest an opposing pattern of neural activation in the vmPFC and dmPFC, indicating that distinct regions within the medial prefrontal cortex are engaged in processing different aspects of social knowledge (Zamboni et al., 2009). The vmPFC seems primarily involved in self-referential processing and is also recruited when processing information about others involves a self-referential evaluation. In contrast, the dmPFC is linked to more universally applicable social-cognitive processes that are independent of self-reference (Zamboni et al., 2009). In this study, statements focusing on individual perspectives engaged the vmPFC because they likely prompted self-referential processing, while statements emphasizing societal interests activated the dmPFC due to their requirement for processing information related to others. Furthermore, the activation of the dlPFC in response to certain statements may reflect its role in deliberative decision-making during complex social evaluations.

### 1.4 Leadership-centric branding

The concept of leader-centric branding, an idea proposed by scholars such as Newman (1994) and subsequently developed by Caprara et al. (2002), further underscores the way political leaders embody their party’s identity. Contemporary neuroscientific methodologies, by offering the tools necessary for an objective measurement of the influence of leadership traits on voter decision-making, have underlined the importance of leader-centric branding (Fisher et al., 2008; Vecchiato et al., 2011). At the core of this unfolding narrative resides the exploration of leadership perception, an influential concept that profoundly shapes voter behavior, the construction of party brand images, and electoral outcomes. Researchers have plumbed the multifaceted reverberations emanating from media portrayals, campaign messaging, and the rapidly evolving socio-digital terrain on which political interactions unfold. However, the vulnerability of traditional methodologies to self-report biases has paved the way for the emergence of political neuromarketing, thereby ushering in a new epoch replete with objective and quantifiable yardsticks to gauge voter responses.

Certain cerebral regions exhibit significantly heightened activation in response to the visage of a political candidate from an opposing party compared to one from the observer’s own political affiliation. These neural activations concern the dlPFC, the insula, and the anterior cingulate cortex (ACC), as well as the supplementary motor area, the cuneus, and the pre-central gyrus (Kaplan et al., 2007). Notably, there is a noteworthy percent signal change for individuals affiliated with different political parties in three key regions—the dlPFC, the ACC, and the insula—where greater activation occurs when viewing the opposing candidate vis-à-vis their own candidate. It was noted that the brain activity elicited while viewing presidential candidate visages is influenced by the political attitudes of observers, and, particularly, the act of viewing the opposing candidate as opposed to one’s own candidate triggers activation in the dlPFC and the ACC (Kaplan et al., 2007). Within the ACC, there exists a subdivision between “emotional” and “cognitive” sectors, with the observed activation predominantly situated in the “cognitive” domain. This specific ACC subregion is linked to attentional control and self-monitoring, and operates in conjunction with the dlPFC to oversee response conflict and engage cognitive control when needed. The activity within this network correlates with individuals’ self-reported emotional reactions to the candidates, with greater dlPFC activation observed when individuals harbor more negative sentiments toward the opponent and more positive sentiments toward their own candidate. This suggests that the images of candidates trigger cognitive control mechanisms for emotional self-regulation (Kaplan et al., 2007).

Neurological activity within brain regions previously associated with evaluative cognitive processes and research pertaining to ideological disparities, such as the insula and the ACC, displayed variations contingent upon the interplay of incongruence, candidate categorization (ingroup or outgroup), and individuals’ political ideology (Haas et al., 2017). Specifically, individuals with more liberal political orientations exhibited increased neural activation when confronted with incongruent as opposed to congruent stimuli, particularly when observing candidates from their own political group (Haas et al., 2017). In other words, liberal-leaning participants were prone to exhibit increased levels of neural activation in the ACC and insula when encountering incongruent stimuli compared to congruent ones, especially when these stimuli featured political candidates affiliated with their own ideological group (Haas et al., 2017). This highlights that the medial prefrontal cortex’s involvement can be extended to regions associated with evaluative cognitive processes and to the detection of cognitive conflicts more broadly, such as the insula and ACC. Furthermore, this neural processing is demonstrably influenced by both an individual’s political ideology and their affiliation with a particular political group.

### 1.5 Methodological transformation: neuroscientific perspective

In Western liberal democracies, the field of political marketing stands at the precipice of transformative change, catalyzed by the rapid march of technological advancements and the burgeoning landscape of data sciences. This paradigm shift is inexorably intertwined with the exigencies of contemporary politics, wherein the imperative for real-time responsiveness to dynamic voter sentiment has assumed paramount significance for political viability. Traditional research methodologies, encompassing established tools such as focus groups and surveys, have burgeoned to assimilate an intricate tapestry of data analytics, machine learning algorithms, and predictive voter modeling. This evolutionary trajectory accentuates the profound interplay between the spheres of political leadership, party branding, and voter behavior. The symbiotic fusion of neuroscientific instruments, most notably functional Magnetic Resonance Imaging (fMRI) and Electroencephalography (EEG), heralds a watershed juncture in comprehending the neural crucible within which political messages undergo processing in the human brain. This stride forward in understanding illuminates the intricate cognitive and emotional circuitry governing the labyrinthine expanse of voter decision-making, thereby spotlighting the alignment between these neural choreographies and the decisions made by individual voters. Of considerable import, the concept of neurobranding plunges into the depths of how branding elements trigger neural responses, in turn influencing voter conduct, thereby underscoring the transformative power inherent in these innovative methodologies.

Traditional approaches to neuropolitics based on neurometrics, such as self-reported measures and fMRI, have several limitations, since self-reported measures are susceptible to social desirability bias, and fMRI is expensive and time-consuming (Pereira et al., 2009). The active use of machine learning algorithms offer several advantages over these traditional approaches. First, machine learning algorithms can be used to analyze large datasets of neuroimaging data, which is not feasible with traditional approaches (Alexandre et al., 2014). This allows researchers to identify subtle patterns in the data that might be missed by traditional methods. Second, machine learning algorithms can be used to develop models that can predict voter behavior or political leader perception based on brain activity, as done in other fields such as student performance and applied neuroscience (Rice and Dean, 2005; Pereira et al., 2009). This outcome is generally not possible when traditional approaches are used. Third, machine learning algorithms can be used to identify the brain regions that are involved in different aspects of political decision-making. This can help researchers to better understand the neural basis of political behavior. Thus, in this study, machine learning algorithms are initially aimed to analyze fNIRS data to predict voters’ perception of political leaders. This approach has several advantages over traditional approaches, since fNIRS is a relatively inexpensive and portable neuroimaging technique, making it more accessible to researchers as well as less susceptible to motion artifacts than fMRI, or in fewer terms, making it more suitable for studying real-world political stimuli (Cui et al., 2011).

### 1.6 New approaches to political neuromarketing

The combined use of advanced neuroimaging methodologies, data analysis techniques, and machine learning methods has profound implications for the future of political neuromarketing strategy. Empirical findings from this study will shed light on the neural correlates that underpin political perception and leader preference, thus providing researchers with insights into the cognitive and emotional constructs that steer voter conduct. The capacity to predict political leader perception through neuroscientific data and machine learning algorithms is a novel means for gauging voters’ responses, thereby facilitating the crafting of targeted and efficacious political strategies. Moreover, the findings of this study underscore the dynamic interplay characterizing sensory attributes and cultural affiliations in shaping political preferences, thus accentuating the need for generating approaches that are capable of addressing diverse voter groups (Breiter et al., 2015).

The observed prefrontal cortex responses to candidates from opposing parties align with prior research demonstrating similar neural activations in response to faces of racial outgroups. However, in the context of political attitudes, it remains unclear whether voters are motivated to suppress their negative feelings toward the opposing party’s candidate. Three potential explanations for these activations are proposed: dlPFC/ACC activity could reflect the suppression of unwanted negative emotions, the suppression of positive feelings toward the opposing candidate, or an up-regulation of negative feelings about the opposing candidate. The dlPFC’s role in regulating emotional responses in general suggests that the activity observed may relate to attempts to intensify negative emotions toward the opposing candidate. Notably, medial prefrontal cortex (mPFC) activity does not signify positive emotion but is activated when making decisions laden with affective content.

The current study aims at understanding the neural activation patterns of voters’ leader perception within the prefrontal cortex and providing a predictive model within this context, as well as developing an objective method for political neuromarketing strategies. Synthesizing the latest findings from the domains of political science, neuromarketing, and decision neuroscience, this research aims to construct a framework that would delineate the contours of responsible practice. In particular, by unifying the evolving trends within political branding and identifying the gaps in the existing academic literature, this interdisciplinary study lays the intellectual groundwork for future empirical inquiries in this rapidly evolving field.

## 2 Materials and methods

### 2.1 Participants

Participants were recruited via targeted invitations sent to members of party district assemblies residing in Istanbul and representing two distinct political parties. A total of thirty-two participants volunteered to take part in the study. To minimize potential gender-related confounding variables, we selected only male participants. The average age of the participants who were also actively engaged in professional activities was 34.76 years (between 25 and 49, *SD* = 6.21). The difference in age distribution of participants from both parties was not statistically significant [*t*(30) = 1.79, *p* = 0.08]. This study was conducted in adherence to the principles outlined in the Declaration of Helsinki (World Medical Association, 2013). After the participants were instructed about the experiment and their questions were answered, they signed informed consent forms before moving on to the data collection phase. Their right to leave the experiment at any moment if they experience any form of discomfort for any reason was also explained. Moreover, the oral and written instructions covered the fact that the collected datasets would not be shared with any third parties. The participants were mainly asked to respond to the given leader–adjective pairings on a three-point Likert scale (Jacoby and Matell, 1971).

### 2.2 Optical brain imaging system

The present investigation used a near-infrared spectroscopy (NIRS) system as primary data collection instrument. The NIRS system utilized in this study is produced by fNIR Devices, model 1100^1^ and is rooted in the research development units of Drexel University (Philadelphia, PA, USA) (Ayaz, 2010). The system consists of three elements: a flexible sensor with 16 optodes (8 light sources and 8 detectors) arranged in a 4 × 4 grid, and which is securely fixed to the participant’s head; the control box with electronic components and analog-to-digital converters; and the system computer, which runs the COBI Studio software and facilitates real-time data monitoring and recording. The sensor, equipped with four distinct light sources, detects oxygenation levels through ten detectors while concurrently recording data streams across sixteen distinct channels (Ayaz et al., 2011). Notably, the sensor is designed so that the light source and the detector are approximately 2.5 centimeters apart, thereby enabling measurements from depths of approximately 1.25 centimeters. This fNIRS system uses two wavelengths (760 nm and 830 nm) to measure the concentration of oxygenated hemoglobin (HbO2) and deoxygenated hemoglobin (Hb) in the blood. Functionally, the system boasts a data acquisition frequency of 2 samples per second (2 Hz) and is capable of measuring neural activity within the Brodmann areas BA9, BA10, BA44, and BA45 (Ayaz et al., 2011).

The non-invasive and portable nature of the optical brain imaging system is instrumental to its effectiveness. Employing a flexible sensor, the system is positioned on the participant’s forehead, facilitating the measurement of changes in neural activation levels within the frontal brain region (Jobsis, 1977). The optical brain imaging system measures oxygenation levels and variations in blood volume within the brain, offering insights into increased functionality (Bunce et al., 2006). Concomitantly, an augmentation of oxygenation levels is anticipated within regions correlated with enhanced cognitive activity via this optical brain imaging system. The system’s cost-effectiveness, security, portability, and relatively high temporal and spatial resolution make it increasingly popular in contemporary brain imaging investigations (Bunce et al., 2006).

### 2.3 Experimental procedure

Data for the study was acquired in accordance with the requirements of the Declaration of Helsinki. COBI Studio Software was used for signal acquisition, and is synchronized with E-Prime Software (v2.0). The latter was used for stimuli presentation with the use of markers sent via parallel port, and fNIRSoft was used for data pre-processing and the first stages of the analyses (Ayaz, 2010). In accordance with the research framework, five positive and five negative adjectives were selected, based on previous field studies conducted in the domain of political marketing, and which were validated by the expert opinions of professionals and scholars (Girişken, 2010). Positive adjectives included “hardworking,” “honest,” “religious,” “leader,” and “powerful,” whereas negative adjectives included “passive,” “lazy,” “weak,” “turncoat,” and “arrogant.” During the experiment, participants were shown these adjectives for 6 s each, displayed beneath an image of either Party A or Party B’s leader. The presentation exposed participants to each pairing individually, followed by the next fixation screen. This resulted in a total of twenty combinations for the two leaders, presented in a randomized sequence. Participants responded using a three-point Likert scale (disagreement, indecision, agreement) for each pairing.

An 8-s fixation screen separated each leader–adjective pair. Throughout the experiment, participants’ prefrontal brain region activations were continuously monitored using optical brain imaging (fNIR). This monitoring aimed to capture real-time neural responses to the presented stimuli, facilitating a direct correlation between the cognitive processing of the image-adjective pairings and the neural activity within the prefrontal cortex. Initially, the fixation screen was presented for 8 s, with the final 4 s serving as the baseline for the upcoming leader-adjective pair. After displaying the leader-adjective pair for 6 s, the subsequent fixation screen was presented, which served as the baseline for the next stimulus.

### 2.4 Data pre-processing

The data was pre-processed in alignment with the temporal dynamics between stimulus exposure and tissue oxygenation. fNIRS data from 16 optodes at two wavelengths was pre-processed using a 20th-order, 0.1 Hz low-pass filter to attenuate high-frequency noise from respiration and cardiac pulsation (Ayaz et al., 2012). Saturated channels, where light intensity at the detector exceeded the analog-to-digital converter limit, were excluded. Motion artifacts were detected and removed using a sliding window motion artifact filter (Ayaz et al., 2011). Rest and task epochs were extracted from the continuous data using time synchronization markers. Blood oxygenation changes (ΔHbO2 and ΔHbR) were calculated for each optode during each block (leader-adjective pair and decision) using the modified Beer-Lambert Law with respect to a resting baseline oxygenation at the beginning of each trial (Ayaz, 2010). Following this pre-processing, each pairing phase was subtracted from the mean activity observed during the preceding resting screen, thereby accentuating the effect of the stimulus. Ultimately, the mean value for each pairing phase was computed, enabling subsequent comparison. The 6-s trials were then averaged to form evoked oxygenation signals (ΔHbO2 minus ΔHbR). Finally, these evoked signals were labeled according to the participants’ decisions, and these labels were utilized as the target classes for subsequent data analyses.

Our primary dataset encompassed a set of 640 observations (derived from 32 participants responding to 20 stimuli) across 19 attributes. Central to our study were the columns representing the “Participant,” “Party Affiliation,” “Response Code,” and the tissue oxygenation values captured from 16 distinct optodes. The pivotal “Response Code” served as a reflection of the participants’ sentiments toward certain party leaders, categorizing them into positive or negative groups. The positive group is formed from responses where the participant does not agree with the pair, and the negative group is formed from responses where the participant agrees with the pair. The “Null” values in responses originated from instances where the participant responded as indecisive, chose not to respond or could not respond within the given duration. These 20 observations with “Null” values were removed to maintain data integrity. In our pre-processing steps, any row containing more than 33.3% of missing values was subjected to a stringent rule: all values within such rows were converted to Nulls, thereby facilitating their exclusion. Data from 32 participants were initially collected, but due to excessive artifacts, the data from one participant was excluded from the dataset during the pre-processing stage.

Outlier detection within each participant’s data was conducted utilizing a z-score method, calibrated against the computed mean and the standard deviation. Values surpassing a z-score of 4 threshold were amended to the closest boundary value, with this correction applied distinctly for each “Response Code” category. An KNN Imputer method was then implemented for the imputation of missing data. KNN imputer algorithm functions by estimating missing values in a dataset based on the k-nearest neighbors, using their value as a reference to infer the missing data (Zhang, 2010). Variance normalization across measurement columns was achieved through the application of the standard scaler algorithm, ensuring a normalized range of data on an individual basis. This algorithm centers each feature by subtracting the mean and scaling it to unit variance, thus standardizing each feature to a mean of zero and a standard deviation of one (Pedregosa et al., 2011).

A stratified holdout method (500 observations for the training and 120 testing) was used by selecting 6 random participants. The hold-out method is a widely used validation technique in machine learning that involves setting aside a subset of the available data to evaluate the performance of a model without using this subset during the training phase. By selecting participants, this method ensures that the participant’s data used for evaluation remains independent from the data used for training, providing a more accurate assessment of the model’s generalization ability (Hastie et al., 2009). During the model development phase, a fivefold cross-validation was applied to the training dataset (Vabalas et al., 2019; Baumgartl et al., 2020).

### 2.5 Model development

The subsequent phase investigated the effects of diverse machine learning algorithms on the pre-processed dataset. This exploration included steps such as model selection, hyperparameter tuning, training, and rigorous evaluation, facilitating a thorough juxtaposition of algorithmic performance. The algorithms applied to the dataset included: Logistic Regression (Pampel, 2021), Random Forest (Breiman, 2001), k-Nearest Neighbors (k-NN) (Hjaltason and Samet, 1999), Decision Tree (Dietterich, 2000), AdaBoost (Bühlmann and Hothorn, 2007), XGBoost (Chen and Guestrin, 2016), Extra Tree Classifier (Geurts et al., 2006), CatBoost (Dorogush et al., 2018), LightGBM (Lin et al., 2022), and Stacking Classifier (Džeroski and Ženko, 2004). Each of these algorithms with distinct characteristics, are frequently utilized in studies focusing on predictive models, particularly in the realm of neuroscience. This can be seen in the work of Glaser et al. (2019), which provides a comprehensive review of machine learning applications in neuroscience. The methodologies outlined in Table 1 elucidate the optimization of algorithmic performance and contribute to the selection of the appropriate models for distinct analytical tasks. Machine learning algorithms are useful tools to analyze data obtained when investigating decision-making processes.

**TABLE 1:** The machine learning algorithms used for model development.

Machine learning algorithm	Strengths	Weaknesses
Logistic regression	– Simple, interpretable linear model. – Fast to train and predict. – Effective for binary classification problems. – Outputs well-calibrated predicted probabilities.	– Assumes linear relationship between predictors and target. – Can be outperformed by complex models on non-linear data. – Sensitive to irrelevant features and outliers. – Not suitable for complex relationships without feature engineering.
Random forest	– Robust against overfitting due to ensemble approach. – Handles high-dimensional data well. – Suitable for both classification and regression tasks. – Provides feature importance ranking. – Resistant to noise and outliers.	– May require careful tuning of hyperparameters. – Can be computationally intensive for large datasets. – Interpretability of individual trees might be challenging. – Prone to bias in favor of dominant classes. – Difficult to visualize complex interactions.
K-nearest neighbors	– Intuitive and easy to understand. – Non-parametric nature accommodates complex decision boundaries. – Robust to noisy data. – No assumptions about data distribution. – Can be adapted for both classification and regression tasks.	– Sensitive to the choice of k (number of neighbors). – Computational complexity increases with large datasets. – Can be affected by irrelevant features. – Poor performance if data has varying densities. – Doesn’t handle high-dimensional data efficiently.
Decision tree	– Easily visualized and interpreted. – Can handle both numerical and categorical data. – Does not require data normalization. – Can model non-linear relationships.	– Prone to overfitting, especially with deep trees. – Can be unstable with slight data changes. – Biased with imbalanced datasets.
AdaBoost	– Boosting technique improves weak learners. – Less prone to overfitting. – Aggregates results for improved accuracy. – Adapts quickly to changes in the data.	– Sensitive to noisy data and outliers. – Requires careful tuning of hyperparameters.
XGBoost	– Powerful ensemble method with high predictive accuracy. – Handles missing data effectively. – Regularization and pruning prevent overfitting. – Supports various evaluation criteria. – Handles imbalanced classes through weighted sampling.	– Prone to overfitting if hyperparameters are not properly tuned. – Requires careful selection of learning rate and tree-specific parameters. – Can be computationally intensive. – Black-box nature makes interpretation challenging. – Potential for biased predictions if not balanced properly.
Extra tree classifier	– Random splits lead to reduced variance. – Generally faster than Random Forest due to randomness. – Can be less prone to overfitting.	– Might be less accurate than random forest. – Random splits can sometimes produce suboptimal trees.
CatBoost	– Handles categorical data directly. – Less prone to overfitting with default parameters. – Built-in support for missing data. – Has an efficient implementation.	– Can be slower to train compared to other GBMs. – Parameter tuning can be complex for novice users.
LightGBM	– Fast training and efficient memory usage. – Supports categorical features. – Suitable for large datasets with improved accuracy. – Uses gradient-based one-side sampling.	– More sensitive to overfitting with small datasets. – Requires careful tuning for optimal performance. – Might be less intuitive than traditional GBMs.
Stacking classifier	– Utilizes multiple layers of learning for enhanced accuracy. – Leverages diverse model types for improved generalization. – Can capture complex patterns through hierarchical approach. – Integrates multiple viewpoints for more robust predictions. – Potential for discovering nuanced relationships.	– Complex to implement and tune due to multiple layers. – Prone to overfitting if not carefully validated. – Requires substantial computational resources for training. – Risk of information leakage between layers. Interpretability challenges arise from multi-level architecture.

Hyperparameter tuning was conducted to optimize our machine learning models, focusing on parameters such as learning rate, number of trees, and maximum tree depth (Hernández-Lobato et al., 2017). Hyperparameter finetuning is important because the performance of a machine learning model can be highly sensitive to the values of its hyperparameters (Hutter et al., 2019). We employed a grid search approach, systematically evaluating the model’s performance across various hyperparameter settings on a fivefold cross validation over the held-out training set (Coelho and Silva, 2018). This approach helped identify the most effective hyperparameter values for our final model training.

Selecting the suitable algorithms and configuring their hyperparameters accordingly has a significant influence on the effectiveness of the models. In this study, we considered a large selection of algorithms, each taking a distinct approach to classification via different parameter values and the best resulting parameters were shown in Table 2. Through the rigorous evaluation of their performance, valuable insights have been obtained as to their effectiveness across a spectrum of scenarios. The ensuing sections delineate the step-by-step approach undertaken to assess these machine learning algorithms.

**TABLE 2:** Parameters used during model development.

Algorithm	Critical parameters used during model development	Tested range/values
Logistic regression	C: This parameter controls the strength of the L2 regularization. A higher value of C will lead to a more regularized model, which may be less accurate but more robust to overfitting.	(0.01, 0.02, 0.03, 0.05, 0.06, **0.07**, 0.08, 0.09, 0.1, 0.2, 0.5, 1, 2, 3, 5)
	Penalty: this parameter is used to specify the norm used in penalization.	l2, elasticnet, none, l1
	Class_weight: this parameter weights associated with classes.	**Balanced**, none
	Solver: this parameter specifies the algorithm to use in the optimization problem.	lbfgs, **liblinear**, newton-cg
Random forest	n_estimators: this parameter controls the number of trees in the forest. A higher number of trees will lead to a more accurate model, but it will also take longer to train.	(**100**, 200, 300, 4000)
	Max_depth: This parameter controls the maximum depth of each tree in the forest. A deeper tree will be able to learn more complex relationships between features, but it will also be more prone to overfitting.	(**3**, 5, 6, 7, 8, 9, 10)
K-nearest neighbors	n_neighbors: this parameter controls the number of neighbors to use to make a prediction. A higher number of neighbors will make the model more robust to noise, but it may also reduce the accuracy of the model.	(1, 2, 3, 4, 5, 6, 7, **8**, 9, 10, 11, 13, 15)
	Weights: this parameter controls the weights that are given to the different neighbors when making a prediction. The default weight is “uniform,” which means that all neighbors are given equal weight. Other possible weights include “distance” and “inverse distance.”	Uniform, **distance**
	Algorithm: this parameter controls the algorithm that is used to find the nearest neighbors. The default algorithm is “auto,” which will automatically select the best algorithm based on the data.	**Auto**, ball_tree, kd_tree, brute
Decision tree	Max_depth: this parameter controls the maximum depth of the tree. A deeper tree will be able to learn more complex relationships between the features, but it will also be more prone to overfitting.	(3, **4**, 5, 6, 7, 8, 9, 10, 11, 12, 13, 15)
	Max_features: this parameter defines the number of features to consider when looking for the best split.	**Auto**, sqrt, log2
AdaBoost	n_estimators: this parameter controls the number of base learners in the ensemble. A higher number of base learners will lead to a more accurate model, but it will also take longer to train.	(100, **200**, 300, 400)
	Learning_rate: this parameter controls the learning rate of the AdaBoost algorithm. A higher learning rate will make the algorithm learn more quickly, but it may also make it more prone to overfitting.	(0.01, 0.03, 0.05, 0.06, 0.07, **0.08**, 0.09, 0.1, 0.2, 0.3, 0.5, 0.6, 0.7, 0.8, 0.9, 1, 1.4, 1.5)
XGBoost	n_estimators: this parameter controls the number of trees in the forest. A higher number of trees will lead to a more accurate model, but it will also take longer to train.	(100, **150**, 200, 250)
	Max_depth: this parameter controls the maximum depth of each tree in the forest. A deeper tree will be able to learn more complex relationships between the features, but it will also be more prone to overfitting.	(**3**, 4, 5, 6, 7, 8)
	Learning_rate: This parameter controls the learning rate of the XGBoost algorithm. A higher learning rate will make the algorithm learn more quickly, but it may also make it more prone to overfitting.	(0.01, 0.04, 0.05, **0.06**, 0.07, 0.08, 0.1, 0.3, 0.5, 0.6, 0.7, 0.8, 0.9, 1.0)
Extra tree classifier	n_estimators: this parameter controls the number of trees in the forest. A higher number of trees will lead to a more accurate model, but it will also take longer to train.	(100, 200, 300, 400, 450, **500**, 550)
	Max_depth: this parameter controls the maximum depth of each tree in the forest. A deeper tree will be able to learn more complex relationships between the features, but it will also be more prone to overfitting.	(3, 4, 5, 6, 7, **8**, 9, 10, 11, 12, 13, 14)
CatBoost	Iterations: this parameter controls the number of iterations the algorithm will run. learning_rate: this parameter controls the learning rate of the CatBoost	(**50**, 100, 150, 200)
	Depth: this parameter controls the depth of the tree. A deeper tree can model more complex relationships, but may lead to overfitting.	(2, **3**, 4, 5, 6)
	Early_stopping_rounds: if the model’s performance does not improve after a specified number of rounds, the training will be halted early to prevent overfitting.	(**1**, 2, 3, 4, 5, 6, 7, 8, 9)
	Learning_rate: this parameter determines the step size at each iteration while moving toward a minimum of the loss function. A lower value will make the optimization more robust, but the convergence will be slower.	(0.01, 0.03, 0.05, 0.1, 0.3, 0.5, 0.7, **0.8**, 0.9, 1, 1.3)
Light GBM	Boosting_type: this parameter defines the type of algorithm to run. “gbdt” stands for gradient boosting decision tree, “goss” stands for gradient-based one-side sampling, and “dart” stands for dropouts meet multiple additive regression trees.	gbdt, goss, **dart**
	Learning_rate: this is the rate at which the model corrects for errors from the previous iteration. A smaller learning rate can lead to a more accurate model but will take longer to train.	(0.01, 0.03, 0.05, 0.1, 0.3, 0.5, 0.6, **0.7**, 0.8, 0.9, 1, 1.5)
	Num_iterations: specifies the number of boosting iterations, which corresponds to the number of trees added to the model.	(50,**100**, 150, 200)
	Early_stopping_rounds: if the model’s performance does not improve after a specified number of rounds, the training will be halted early to prevent overfitting.	(**1**, 2, 3, 4)
	Max_depth: this parameter controls the maximum depth of each tree in the forest. A deeper tree will be able to learn more complex relationships between the features, but i will also be more prone to overfitting.	(2, **3**, 4, 5, 7)

The bold values represent the best resulting parameters for each algorithm and parameter set evaluated in our study. These values signify the optimal configurations identified through our analysis, providing clear indications of the most effective parameter choices within the tested range for each respective algorithm.

### 2.6 Evaluation of the machine learning models

The performance of the machine learning models will be evaluated on a held-out validation set to assess their generalizability. The validation set will consist of 20% of the participants who were not included in the training set. The standard metrics of accuracy, precision, recall, and F1 score are used to evaluate the performance of the models; they are presented in Table 3.

**TABLE 3:** Evaluation metrics for machine learning models.

Term	Definition	Formulation
Accuracy	The proportion of predictions that are correct.	(True positives + True negatives)/Total
Precision	The proportion of positive predictions that are correct.	True positives/(True positives + False positives)
Recall	The proportion of actual positive cases that are correctly predicted.	True positives/(True positives + False negatives)
F1 Score	A harmonic mean of precision and recall.	2 × (Precision × Recall)/ (Precision + Recall)

The Area Under the Curve (AUC) is a critical metric for assessing binary classifiers. It is derived from the Receiver Operating Characteristic (ROC) curve, which illustrates the model’s true positive rate (sensitivity) against its false positive rate (1–specificity) across different thresholds (Witten et al., 2016). Essentially, the AUC, ranging from 0 to 1, evaluates the model’s ability to correctly classify positive and negative cases, with 1 indicating perfect accuracy and 0.5 denoting a random chance level of accuracy. This metric is also valuable in understanding voter behavior for its robustness against varying class distributions in the data.

### 2.7 Explainability of the machine learning models

The model explainability is generally assessed using a variety of techniques including feature importance, partial dependence plots, and counterfactual explanations (Samek et al., 2017). The feature importance method measures the importance of each feature in the predictive model. The feature importance will be calculated using the permutation importance method which involves randomly permuting each feature in the training set and observing the change in the model’s performance (Samek et al., 2017). The features that have the largest impact on the model’s performance are considered to be the most important ones.

The SHAP (SHapley Additive exPlanations) method is a model-agnostic approach to explaining the predictions of machine learning models; it is based on the Shapley values, a concept from game theory that measures the contribution of each player to a coalition (Wang et al., 2021). In the context of machine learning, the SHAP value of a feature is the average difference between the model’s prediction with and without the feature, and these SHAP values calculated for all features can be added up to explain the model’s prediction for a given input (Wang et al., 2021). The SHAP method has several advantages over other methods for explaining machine learning models as it is considered to be model-agnostic, consistent, and locally interpretable (Doshi-Velez and Kim, 2017).

The model’s predictions are calculated for all possible subsets of the features and then the average difference between the model’s prediction with and without a given feature is calculated for each subset (Wang et al., 2021). The SHAP value for a given feature is calculated as the average of these differences. The SHAP values can be used to explain the model’s predictions in a variety of ways. For example, they can be used to generate waterfall plots, which show the impact of each feature on the model’s prediction. SHAP values can also be used to identify the features that are most important for the model’s predictions.

## 3 Results

### 3.1 Model performance outputs

After the intensive period of model development, the model evaluative metrics were generated and presented as tables, and each of these has been investigated in more detail. Below, the best models with respect to the conducted grid search optimization are presented. Of all these models, LightGBM appears to be the one with the highest performance (Table 4).

**TABLE 4:** Classification report for the predictive models.

Model	Accuracy	Precision	Recall	F1	AUC
Logistic regression	0.750	0.750	0.750	0.750	0.812
Random forest	0.725	0.725	0.725	0.725	0.854
KNN	0.758	0.758	0.758	0.758	0.791
Decision tree	0.700	0.701	0.700	0.700	0.765
Ada boost	0.734	0.734	0.734	0.733	0.858
XGBoost	0.767	0.768	0.767	0.767	0.880
Extra tree	0.750	0.750	0.750	0.750	0.849
Cat boost	0.775	0.775	0.775	0.775	0.882
LightGBM	0.784	0.788	0.784	0.783	0.883
Stacking	0.775	0.776	0.775	0.775	0.867

As shown in Table 4, LightGBM outperforms all other models on all metrics. It demonstrates an accuracy of 0.78, a precision of 0.79, and a recall of 0.78. The F1 score, representing the harmonic mean of precision and recall, is also 0.78. Furthermore, the model achieves an AUC of 0.88. These values make LightGBM the best model for predicting voters’ perceptions of political leaders. We can also see from Table 4 that all the models perform well on the data. This suggests that the data is informative and that the task of predicting voter perceptions of political leaders is feasible. Overall, the model performance outputs are very positive. These results suggest that the proposed approach of using machine learning algorithms to analyze fNIRS data to predict voter perceptions of political leaders is promising.

### 3.2 Model explainability

The models formulated on the basis of the fNIRS measurements provide compelling information about the participants’ adverse reactions to leader–adjective congruence. It is a crucial fact that fNIRS signals are not always directly associated with specific brain regions, and that there can be variability in how signals are interpreted across different studies. This is due to the fact that fNIRS measures changes in blood oxygenation levels, which can be influenced by a variety of factors, including blood flow, metabolism, and brain activity. Despite these limitations, fNIRS can be a valuable tool for studying brain activity, particularly in situations where other methods, such as fMRI, are not feasible.

Notably, the majority of negative responses are associated with specific neural activations. Optode 13, which corresponds to the right dorsolateral prefrontal cortex (dlPFC), exhibits notable oxygenation (Oxy) measurements indicative of negative responses. Similarly, the right dorsomedial prefrontal cortex (dmPFC), measured by optode 9, and the left ventromedial prefrontal cortex (vmPFC), measured by optode 8, exhibit notable oxygenation (Oxy) measurements indicative of increased Oxy levels correlated with negative responses. Further noteworthy patterns, which exhibit notable oxygenation (Oxy) measurements indicative of positive responses, emerge from the following: optodes 7, corresponding to the left dmPFC; optode 10, corresponding to the right vmPFC; optode 11, corresponding to the right dmPFC; optodes 14 and 15, which correspond to the right dorsolateral prefrontal cortex (dlPFC); and optode 1, corresponding to the left dlPFC.

**FIGURE 1:**
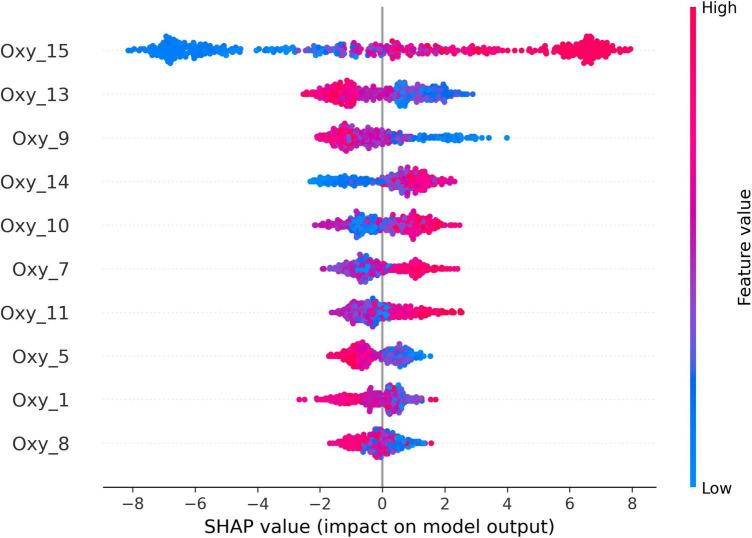
SHapley Additive exPlanations (SHAP) output of the XGBoost model.

The SHAP output of the XGBoost model (Figure 1) shows the relative importance of each feature in predicting the participants’ reactions to leader–adjective congruence. The features are ranked in order of importance, with the most important features at the top. As shown in Figure 1, the most important features for predicting negative reactions are acquired from the optodes 13, 9, and 8, which correspond to the right dlPFC, right dmPFC, and left vmPFC, respectively. This is consistent with the findings described above, which suggest that these brain regions are involved in processing leader–adjective congruence and generating negative responses. Other important features for predicting negative reactions include optodes 7, 10, 11, 14, and 15, which correspond to the left dmPFC, right vmPFC, right dmPFC, right dlPFC, and left dlPFC, respectively. These findings suggest that these brain regions are also involved in processing leader—adjective congruence and generating negative responses.

### 3.3 Evaluation of model fairness

The fairness of the developed models was evaluated by comparing the model outcomes for party A and party B voters separately. The classification reports for these party voters are presented in Tables 5, 6 below. As shown in Tables 5, 6, the model performance for party A and party B voters is very similar. The precision, recall, F1 score, AUC, and ROC curve are all very high for both groups. This suggests that the model is not biased toward either party.

**TABLE 5:** Evaluative metrics for the members of party A.

	Precision	Recall	F1 score
0 (negative)	0.79	0.77	0.78
1 (positive)	0.77	0.80	0.79
Accuracy			0.78
Macro average	0.78	0.78	0.78
Weighted average	0.78	0.78	0.78

**TABLE 6:** Evaluative metrics for the members of party B.

	Precision	Recall	F1 score
0 (negative)	0.70	0.72	0.71
1 (positive)	0.73	0.71	0.72
Accuracy			0.72
Macro average	0.72	0.72	0.72
Weighted average	0.72	0.72	0.72

The last two rows of Tables 5, 6 refer to macro average and weighted average. For the macro average the sample sizes of the classes are not taken into consideration but only their average values of the classes are calculated whereas for the weighted average, the averages values are weighted with respect to the class sizes. Since the support values for these classes are equal to each other, we expect these values to be the same for macro and weighted average values (0.78 for Table 5 and 0.72 for Table 6).

The bias auditing also indicates that there is no significant difference between the two sides. The distribution of the model performance is highly similar for both groups, which further supports the conclusion that the model is not biased. The difference in model performance between party members might be due to the relatively limited sample size. With a larger sample size, it is possible that some small differences in model performance between the two groups would become statistically significant. However, the current results suggest that the model is generally fair and can be used to predict voter perceptions of political leaders for both party A and party B voters.

## 4 Discussion

This study used neuroscientific tools to collect data and machine learning algorithms to analyze fNIRS data in order to predict voter perceptions of political leaders. This approach has the potential to provide new insights into the neural basis of political decision-making and to develop more effective political marketing campaigns. It also has several advantages over traditional approaches to neuropolitics, including the ability to analyze large datasets of neuroimaging data, develop predictive models, and identify the brain regions involved in political decision-making. The findings from this study indicate that members of two opposing political parties demonstrate distinctive neural patterns toward their party leader as opposed to the rival party leader. These distinctive characteristics in neural patterns have been validated using machine learning algorithms and used to provide data-driven predictive models. Overall, the model performance outputs are generally high. The best model, LightGBM, achieves an accuracy of 0.78, a precision of 0.79, a recall of 0.78, an F1 score of 0.78, and an AUC of 0.88. These results suggest that the proposed approach—the use of machine learning algorithms to analyze fNIRS data to predict voter perceptions of political leaders—is promising. The model explainability analysis provides compelling insights into the neural basis of negative reactions to leader–adjective congruence. The findings suggest that the dlPFC, dmPFC, and vmPFC are involved in processing leader–adjective congruence and generating negative responses. These findings have potential implications for the development of more effective political marketing campaigns despite the limitations of the current study, such as the limited sample size, the use of single modality, and the interpretability of the models.

### 4.1 Predictive modeling of the party leader perception

Following a comprehensive assessment of the neural activity taking place during party leader perception when accompanied by different adjectives, this study investigates predictive modeling through the application of machine learning algorithms on neuroscientific data. A diverse array of algorithms, including Logistic Regression, Random Forest, k-Nearest Neighbors (k-NN), Decision Tree, AdaBoost, XGBoost, Extra Tree Classifier, CatBoost, Light GBM, and Stacking Classifier, have undergone a rigorous evaluation to determine their effectiveness in predictive brand perception modeling. These algorithms use fNIRS-derived hemodynamic response data, participant feedback, and communal perception labels to formulate predictive models. The phase of predictive modeling reinforces the intricate interactions between neural activations and brand perceptions. Machine learning algorithms use the complex neural activations measured by fNIRS, coupled with a series of relevant attributes, to predict brand perceptions as positive, negative, or neutral. Model performance evaluation metrics, including F1 score and weighted accuracy, are examined, thereby improving the capability of machine learning to decipher the neural signatures of political leader preference (Critchley et al., 2000; Crosson et al., 2010; Nebe et al., 2023).

The findings obtained in this study suggest that the dlPFC, dmPFC, and vmPFC are involved in processing leader–adjective congruence and generating negative responses. The dlPFC is associated with higher-order cognitive functions, such as working memory, attention, and decision-making. The dmPFC is involved in social cognition, such as understanding and responding to the emotions of others. The vmPFC is involved in emotional regulation and decision-making under uncertainty. These findings are consistent with previous research on the neural basis of political decision-making. For example, a study by Knutson et al. (2006) found that the brain regions including vmPFC and anterior prefrontal cortices are activated during political attitude inducement. The empirical results of a study by Xia et al. (2015) indicate that lateral orbitofrontal cortex (OFC) seems to be responsible for processing information such as candidates’ social competence and attractiveness in political decision-making. Another study by Leong et al. (2020) found that the dmPFC is activated when people process political information that is incongruent with their beliefs. The findings of the present study also have implications for the development of more effective political marketing campaigns. By understanding the neural basis of negative reactions to leaders–adjective congruence, political campaigners can develop strategies to avoid triggering these reactions. For example, they can avoid using incongruent adjectives to describe their candidates or their opponents.

### 4.2 Neural activation patterns and party leader perception

Party leader perception, itself an important part of voting behavior, results from a dynamic interplay of cognitive and emotional processes. This study’s findings reveal the central role assumed by various brain regions, with a particular emphasis on the prefrontal cortex (PFC), in the construction of leader perceptions. Notably, the PFC, involved in higher-order cognitive functions such as decision-making, emotional regulation, and social cognition, plays a key role in orchestrating the neural intricacies of brand perception since the party leaders appear as models for their voters, as if they were brand ambassadors for their political party. The examination of the fNIRS measurements obtained from participants’ responses to leader–adjective compatibility reveals that the hemispheres of the brain are linked to positive and negative emotions without any distinction like frontal alpha asymmetry (Metzen et al., 2022). Additionally, the prefrontal cortex regions exhibit distinct neural activity patterns for leader perception and verbal output. The dorsolateral prefrontal cortex (dlPFC) plays a central role in mediating cognitive conflict during preference formation, underscoring the interplay between cognitive and affective dimensions during brand evaluation (Seminowicz and Moayedi, 2017). Further empirical findings from the relevant literature indicate that the hippocampus and the dlPFC exhibit increased activation when engaging with familiar brands, accentuating the interwoven nature of memory associations and articulated preferences (Deppe et al., 2005). The activity of both the hippocampus and the dlPFC underlines the role of personal history in shaping decisions.

In addition to these, certain areas of the brain show notably increased neural activity when individuals look at the face of a political candidate from a different party, as opposed to a candidate from their own party. The dlPFC and the ACC are more active when voters compare the opposing candidate to their own candidate. Furthermore, the left frontal activity correlates with positive ratings (Kaplan et al., 2007). Together with its role in the manipulation of cognitive representations in the domains of working memory and reasoning, in particular, the left side is crucial for processing verbal and spatial information within working memory, whereas the right side is more dominant for verbal and spatial reasoning (Barbey et al., 2013). Current findings suggest that the left dlPFC (optode 1) is correlated with negative participant responses. On the other hand, increased activity in the right dlPFC (optodes 14 and 15) is a positive response indicator, whereas increased activity in the right dlPFC (optode 13) aligns with negative responses. The right dlPFC is an important brain region for behavioral adaptation in the presence of conflict, and stereotypes and conservatism are linearly associated with increased activation in the right dlPFC (Zamboni et al., 2009). Its involvement in verbal reasoning suggests it influences positive responses by deciphering the congruence between political leader and corresponding adjective; however, another optode at the right dlPFC is correlated with negative responses, which may be caused by exhibiting adaptive behavior in a conflict situation. Another interesting finding with positive responses is the increased activity of the right dlPFC, which might indicate that the majority of the participants were conservative. This activation underscores the potential utilization of the right dlPFC for predictive leader–adjective compatibility, encouraging us to give it a more prominent role within the experimental design.

The dorsomedial prefrontal cortex (dmPFC), another important region for perception, plays a pivotal role in context-based information generation, emotion regulation, and social information processing. It engages in high-construal abstraction during preference evaluations across fields, using task-independent mechanisms to calculate relative subjective value. The empirical findings obtained suggest that increased neural activity in the right dmPFC (optode 9) has potential implications as indicators of negative responses. Conversely, the dmPFC’s role in conflict monitoring signifies that the right dmPFC’s activity pattern might reflect contradictions between party leader and corresponding adjective (Rushworth et al., 2004). Increased activation of the right dmPFC has been associated with community-related situations in voters (Zamboni et al., 2009). On the other hand, increased neural activity in the right dmPFC (optode 11) and in the left dmPFC (optode 7) correlated with positive responses. This activity pattern may be due to the participants’ evaluation of their own party leaders and the leader of the opposing party, in line with socialization. The dmPFC regulates the activity of the dlPFC in carrying out strategic planning within decision-making (Taren et al., 2011). The findings of the current study are the outcome patterns of the right dmPFC and dlPFC. The increased activity in the right dlPFC (optode 13) is associated with negative responses, whereas the increased activity in the right dmPFC (optode 11) is associated with positive responses. The two leaders used in the experiment represent two different views in Türkiye, and the participants are equally split in terms of whose leader they support; thus, the observed pattern may be due to the emergence of adaptive behavior as a result of internal conflict and strategic decision-making efforts in these regions. Still, the activity pattern of the right dmPFC (optodes 9–11) is a potentially promising indicator for understanding and predicting scenarios with altruism.

### 4.3 Value-based decisions prompted by the vmPFC

Political choices, in a sense, are value-based decisions, for which the vmPFC is a crucial region (Krastev et al., 2016). Greater emphasis on individual-related statements is correlated with increased vmPFC activation; moreover, vmPFC is crucial in the processing of self-referential information and is also engaged in the self-referential appraisal of information about others (Zamboni et al., 2009). Neuroscience research has underscored the vmPFC’s predictive capabilities in scenarios based exclusively on sensory cues. The interplay between the vmPFC and the hippocampus–dlPFC–midbrain pathways influences preferences through sensory and cultural information (McClure et al., 2004). The increase in vmPFC activity might indicate the processing of associative information about politicians by activating stereotyped information (Knutson et al., 2005, 2007). Evidently, the effect of the right vmPFC (optode 10) on positive responses stems from an evaluation of the consistency of the leader–adjective pairings, corroborating the present study’s findings. In light of these considerations, the vmPFC emerges as a pivotal region for predicting participants’ perceptions of leaders.

### 4.4 Comparison with earlier studies

Past research on the neural basis of political decision-making has used a variety of neuroimaging techniques, including functional Magnetic Resonance Imaging (fMRI) and Electroencephalography (EEG). However, these techniques have several limitations. fMRI is expensive and requires participants to remain still in a large scanner, which can be difficult for some people (Poldrack et al., 2011). EEG is more portable and affordable than fMRI, but it is also more susceptible to noise and artifacts (Luck, 2014). Functional near-infrared spectroscopy (fNIRS) is a relatively new neuroimaging technique that is less expensive, more portable, and more motion-tolerant than fMRI and EEG (Obrig et al., 2000; Sakatani et al., 2007). fNIRS measures changes in blood oxygenation levels in the brain using near-infrared light. This makes it a well-suited technique for studying brain activity in response to political stimuli, which can be dynamic and involve movement (Cui et al., 2011; Liu et al., 2015). Machine learning algorithms have also been used in previous research on political neuromarketing. However, most of this research has focused on predicting voter behavior based on self-reported measures of political attitudes and preferences (Kosinski et al., 2017; Kehret-Ward et al., 2018). Self-reported measures are often biased and inaccurate, as people are not always aware of their own motivations and biases (Nisbett and Wilson, 1977). The current study used machine learning algorithms to predict voter perceptions of political leaders based on their brain activity.

### 4.5 Limitations of the study

There are several potential limitations related to this study including a relatively small sample size, the use of a single neuroimaging modality, the degree of interpretability of the obtained data, and the gender bias. First, the study was conducted with a relatively small sample of participants (*n* = 31) which limits the generalizability of the findings to the population as a whole, despite the fact that neuroimaging studies are usually conducted with a similar number of participants (Poldrack et al., 2011). Secondly, although we employed a hold-out method and cross-validation to mitigate the risk, it is important to acknowledge that the possibility of overfitting still exists when using machine learning models. Thirdly, the study used only the fNIRS method to measure brain activity. Other neuroimaging techniques, such as EEG and MEG, can provide different types of information about brain activity patterns. Using multimodal neuroimaging techniques would have provided a more complete picture of how voters’ brains respond to political stimuli (Logothetis, 2012). The fourth issue concerns the interpretability of the results: since there is no one-to-one direct correlation between brain activity and brain behavior, the interpretation of the results is not always straightforward. While the results of this study are suggestive, it is important to beware of the limitations of fNIRS in interpreting these results. In order to address this limitation, some studies have used inverse problem solutions to map fNIRS signals onto brain regions (Tremblay et al., 2018; Condy et al., 2021). Inverse problem solutions are mathematical algorithms that use information about the physics of light propagation in the brain to estimate the source of fNIRS signals. However, inverse problem solutions are not without their own limitations, and they can be sensitive to noise and artifacts in the fNIRS data (Kirilina et al., 2012; Hussain et al., 2023). The last limitation is that all the participants were male, because of practical reasons. Despite these limitations, the study provides valuable insights into the way voters’ brains respond to political stimuli. Future research should address the limitations of this study by conducting larger studies with more diverse samples, using multimodal neuroimaging techniques, and controlling for individual differences.

### 4.6 Potential contributions and future prospects

The empirical findings acquired from the current study make a couple of contributions especially related to the use of machine learning models in the field of applied neuroscience, such as targeting specific brain regions, evoking specific emotions, segmenting voters, testing visual messages, and improving campaign design. Firstly, political neuromarketing research methods could be used to segment voters based on their brain activity patterns. This segmentation would allow campaigns to tailor their messages to different voter segments in a more effective way. For instance, a political campaign could develop different messages for voters who are more responsive to emotional appeals vs. rational appeals (De Bruyckere et al., 2015). Secondly, political neuromarketing research could be used to test the effectiveness of different campaign messages. This would allow campaigns to identify the messages that are most likely to resonate with voters. For example, a campaign could use neuroimaging to test the effectiveness of different TV commercials before they are aired: one such test revealed that political ads that featured images of candidates smiling and waving were more effective than ads that featured images of candidates looking serious (Cho, 2013). Thirdly, political neuromarketing research could be used to improve the design of political campaigns. For example, campaigns could use neuroimaging to identify the best way to design campaign websites, social media posts, and other campaign materials since it has been empirically shown that people are more likely to engage with political content that is personalized to their interests (Kosinski et al., 2017). Beyond the exciting potential this interdisciplinary field conveys, it is important to note that political neuromarketing research is still in its early stages, and more research is needed to understand how best to apply these findings to political marketing campaigns. However, the findings of political neuromarketing research have the potential to transform the way political campaigns are conducted. By understanding how voters’ brains respond to political stimuli, campaigns can design more effective messages and strategies.

Future work on the topic should tackle three main issues: studies should use larger and more diverse samples, should use multimodal neuroimaging techniques, and should investigate the neural correlates of other political attitudes and behaviors. First, most political neuromarketing studies have been conducted with relatively small, convenience samples. Future research should conduct larger studies with more diverse samples in terms of age, race, ethnicity, gender, and political ideology. This will help to ensure that the findings of political neuromarketing research are generalizable to the population as a whole. Second, most political neuromarketing studies have used fMRI, which is a good technique for measuring brain activity in specific regions. However, other neuroimaging techniques, such as EEG and MEG, can provide different types of information about brain activity. Future research should use multimodal neuroimaging techniques to get a more complete picture of the way voters’ brains respond to political stimuli. Third, political neuromarketing research has primarily focused on investigating the neural correlates of voter decision-making. However, many other political attitudes and behaviors that are of interest, such as political participation, trust in government, or political ideology. Future research should investigate the neural correlates of a wider range of political attitudes and behaviors.

### 4.7 Conclusive remarks

In conclusion, by untangling the neural correlates governing political perception and preference, this interdisciplinary research is an important contribution to the burgeoning field of political neuromarketing. The confluence of cutting-edge neuroimaging techniques and machine learning algorithms unveils the complex interplay between cognitive, emotional, and cultural factors influencing consumer behavior. This study’s findings underscore the importance of key brain regions, including the dlPFC, vmPFC, and dmPFC, in encoding sensory and cultural cues in shaping brand perceptions. Through the use of machine learning algorithms to predict brand preferences, this study offers insights relevant to strategic brand management and marketing strategies. As the field of political neuromarketing continues to evolve, this research can function as a foundation for future attempts at deciphering the intricate neural dynamics driving consumer behavior, thus paving the way for more effective and context-sensitive strategies.

Inextricably linked to the dynamic landscape of political marketing is the relatively new role of branding within contemporary political campaigns. Political parties are increasingly adopting branding strategies analogous to those employed in consumer marketing, leveraging unique selling propositions, brand promises, and meticulous image management to shape voter perceptions and thereby influence electoral choices (Lees-Marshment, 2001; Smith, 2001). The convergence of political branding and neuromarketing has opened new opportunities to understand voter behavior at a neurophysiological level. At the same time, in light of the transformative power these methodologies wield, ethical considerations are paramount. Integral to this discourse is the imperative of comprehending the attitudinal landscape of party assembly members toward their leaders. The present study’s quest to unveil the neural substrates underpinning responses to adjectives both positive and negative in nature, bearing resonance with leadership traits, unfurls through the vessel of neuroimaging tools. Discerning the differential activation patterns across frontal brain regions amid subjects affiliated with distinct political parties elevates understanding regarding the intricate ballet of leadership perception and party affiliations. While not limited to this, the neural activation patterns in the prefrontal cortex might be unveiling different tendencies such as altruistic punishment decisions and the belief in free-will (Çakar et al., 2022, 2023). In essence, the melange of neuromarketing principles with the expansive domain of political science bequeaths a complex tapestry that unravels the labyrinthine intricacies woven around leadership, party branding, and the sinuous ebb and flow of voter behavior. This fusion, a symphonic convergence transcending the boundaries of academia, harbors within it the solemn ethical obligation to navigate the uncharted realms of the human psyche with an equanimity that combines scholarly rigor and sensitivity.

## Data availability statement

The raw data supporting the conclusions of this article will be made available by the authors, without undue reservation.

## Ethics statement

The studies involving humans were approved by the MEF University Ethics Committee (E-47749665-050.01.04-3516). The studies were conducted in accordance with the local legislation and institutional requirements. The participants provided their written informed consent to participate in this study.

## Author contributions

TÇ: Conceptualization, Data curation, Funding acquisition, Investigation, Methodology, Project administration, Resources, Software, Supervision, Validation, Visualization, Writing – original draft, Writing – review and editing. GF: Data curation, Methodology, Validation, Visualization, Writing – review and editing.

## Footnotes

1
https://fnirdevices.com/
